# Urinary levels of dimethoate, bisphenol A and benzo[a]pyrene in first-year students of Hohai University from different geographical regions

**DOI:** 10.1186/s12889-021-11726-4

**Published:** 2021-09-16

**Authors:** Yu-Juan Xu, Hong-Liang Gao, He Liu, Ning-Wei Zhao, Qi Cheng, Fu-Rong Zhang, Juan Ye, Ai-Qing Wang, Yan-Jun Dou, Bei Ma, Feng Zhu, Xian-Lin Xu, Chao-Jun Li, Jing Wu, Ning Shen, Bin Xue

**Affiliations:** 1 Hohai University, Nanjing, 210098 China; 2 Core Laboratory, Sir Run Run Hospital, Nanjing Medical University, Nanjing, 211166 China; 3 General Surgery Department, Sir Run Run Hospital, Nanjing Medical University, Nanjing, 211166 China; 4 Affiliated Hospital of Nanjing University of Chinese Medicine, Nanjing, 210029 China; 5 Medical School of Nanjing University, Nanjing, 210093 China; 6 Affiliated Hospital of Integrated Traditional Chinese and Western Medicine, Nanjing University of Chinese Medicine, Nanjing, 210028 China; 7 Department of Urology, Sir Run Run Hospital, Nanjing Medical University, 109 Longmian Road, Jiangning, Nanjing, 211100 Jiangsu China; 8 State Key Laboratory of Pharmaceutical Biotechnology, Medical School of Nanjing University & Model Animal Research Center, Nanjing University, Nanjing, 210093 China; 9 China Exposomics Institute (CEI) Precision Medicine Co. Ltd, Shanghai, 200120 China

**Keywords:** Dimethoate, Benzo(a)pyrene, Bisphenol A, Exposure, First-year students

## Abstract

**Background:**

The objective of this study was to detect the urinary levels of dimethoate, benzo(a) pyrene (BaP), and bisphenol A (BPA) in first-year Hohai University students with different geographic origins.

**Methods:**

First-morning urine samples were collected from 540 healthy freshmen aged 17 to 19 years. Chemical levels were measured using β-glucuronidase hydrolysis followed by a high-performance liquid chromatography-tandem mass spectrometry-based method. Geometric means (GMs) of these three chemicals are presented by body mass index (BMI) and location in a volume-based and creatinine-standardized way.

**Results:**

GM concentrations of omethoate, BPA and 3-OHBaP were 9.47 μg/L (10.80 μg/g creatinine), 3.54 μg/L (4.04 μg/g creatinine) and 0.34 ng/L (0.39 ng/g creatinine), respectively. The GM concentration of omethoate in males was significantly higher than that in females. The individuals with a BMI higher than 23.9 had higher GM concentrations of omethoate, BPA, and 3-OHBaP. The inhabitants of Southwest China had significantly lower GM concentrations of omethoate, BPA, and 3-OHBaP than those who lived in other locations in China.

**Conclusion:**

The average level of environmental chemical accumulation in freshmen is lower in Southwest China and differs in youth who live in different regions. In addition, obesity is correlated with higher toxin levels in youth.

## Introduction

Pollution of air, water, and food has been increasing as a consequence of global climate change, pesticide misuse, and industry development [1]; thus, the negative effects of environmental pollution on human health have recently become a serious concern. Inevitably, humans are exposed to pollutants such as heavy metals, pesticide residues, polycyclic aromatic hydrocarbons (PAHs), and bisphenol A (BPA) through drinking water, food, dust, and ambient air [2], and exposure to these pollutants is tightly linked to the initiation and progression of multiple diseases [3].

China is one of the largest agricultural countries in the world, with > 300,000 tons of agricultural pesticides used annually [4]. Dimethoate, one of the most commonly used organophosphorus pesticides, is widely used for broad-spectrum control of a wide range of insects, including mites, flies, aphids, and plant hoppers [5]. Overuse of dimethoate can lead to large amounts of residue on fruits, vegetables, and grains [6]. Existing research has confirmed that even very low levels of dimethoate may have adverse health effects that mainly include neurotoxicity [7–9] and potential carcinogenesis [10–12]. The extensive industrial development model has made China fall into an “environmental pollution–economic development” cycle [13]. PAHs, which originate from diverse sources, including petrochemical products and the combustion of fossil fuels, are pervasive pollutants characterized by their hazardous carcinogenic and mutagenic potential, and these pollutants exist not only in the air but also in food and drinking water [14, 15]. Benzo(a) pyrene (BaP) is one of the most studied PAHs classified by the International Agency for Research on Cancer (IARC) as a Group 1 carcinogen [16]. Multiple studies have shown that BaP requires metabolic activation to exert its carcinogenic effects [17, 18]. It was reported that a higher incidence of forestomach tumors was observed when B6C3F1 mice were exposed to BaP via diet in a long-term study [19]. BPA is a synthetic plasticizer, of which more than 8 million pounds are produced worldwide each year, and BPA can be found from plastic bottles and medical devices to the coating of food packages [20]. Likewise, there is growing evidence that BPA may adversely affect human health. Several studies have demonstrated that BPA has negative effects on human reproduction, including female fertility [21], male sexual function [22], sperm quality [23], etc. Moreover, BPA has an impact on gene expression processes, such as the function of enzymatic proteins, which play important roles in fetal development [24]. In addition, an in vitro study showed that metabolic syndromes such as type 2 diabetes, nonalcoholic fatty liver disease, and obesity are also associated with BPA [25].

Different subjects originating from distinct parts of the country may reflect the environmental exposure in their region. It was reported that the blood Pb levels of the populations who live in Wuhan, central China, were lower than those in Beijing [26]. It was also reported that healthy Chinese individuals who live in areas near manganese mines or nonferrous metal mines have a significantly higher urinary manganese level than those who live in other regions [27]. Another study showed that higher urinary levels of As and Cd were observed in the Wuhan population than in populations in other countries [28]. In addition to the geographic distributions, a preliminary study reported that the hair and urinary aluminum levels in obese subjects were 31 and 46% higher than those in the healthy group, respectively [29].

Hohai University (former Hohai Civil Engineering School of China, established in 1915, HHU) is a national key university under the direct administration of the Ministry of Education. As a comprehensive university with at least 20 colleges, this school enrolls more than tens of thousands of students each year from every province in China. Before entering university, the lifestyle of high school students is relatively unitary during the nearly 10-year study period at the place of birth. It should be considered that environmental background may have a significant effect on long-term health effects. It is meaningful to detect the level of environmental chemical and toxin exposures, which could reflect the local environment and impact individual health. In addition, environmental exposure markers of dimethoate [30], BaP [31, 32], and BPA [33] can be easily detected in blood and urine. In this study, we chose 3 kinds of very common pollutants in the Chinese environment to represent the exposure levels of pollutants in young Chinese people aged 17 ~ 19 years.

Therefore, the objective of the present study was to provide baseline information on the levels of dimethoate, BaP, and BPA in urine samples from first-year Hohai University students with different geographic origins and to assess the correlation between the level of pollutant exposure and geographic origin and BMI at baseline. More importantly, this study will facilitate the improvement of the overall health level of Chinese people by advocating for a healthier lifestyle and providing suggestions for environmental protection policies.

## Materials and methods

### Study design and participants

All procedures, including sampling and examination, were performed in agreement with the principles set forth in the Declaration of Helsinki and its later amendments (2013). All examinees were invited to participate and voluntarily took part in the present study. All subjects were informed about the objectives of the study and experimental procedures and signed the informed consent form. The study protocol was reviewed and approved by the Ethical Review Committee of Sir Run Run Hospital, Nanjing Medical University (2019-SR-018).

**Fig. 1:**
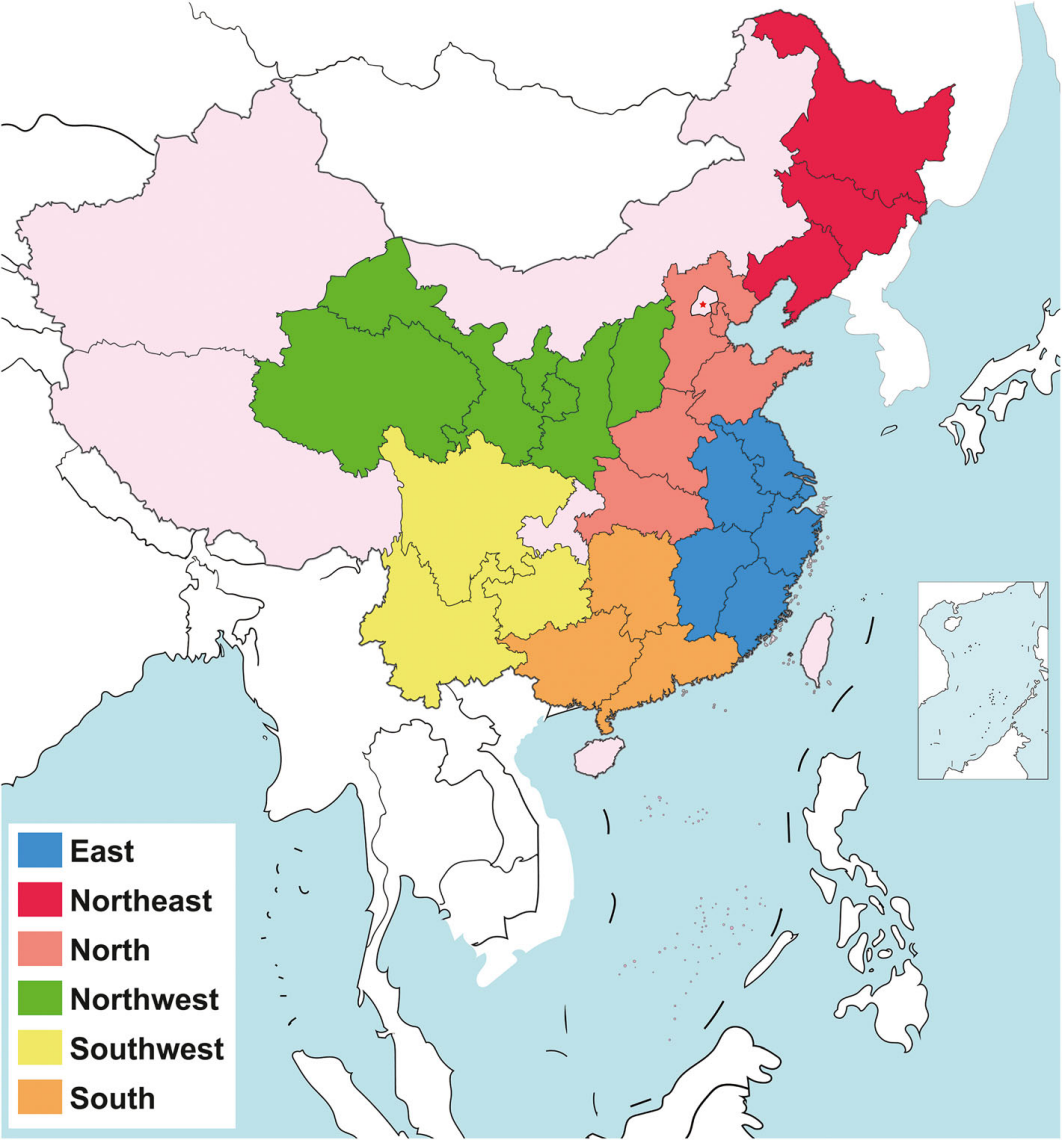
Map of study area showing sampling sites

A total of 540 freshmen attending the HHU originating from East (*n* = 319), Northeast (*n* = 10), North (*n* = 85), Northwest (*n* = 41), Southwest (*n* = 43), and South (*n* = 42) China were enrolled in the present study (Table 1); there were 253 males and 287 females aged from 17 to 19 years. The precise geographical locations in China are shown in Fig. 1.

**Table 1:** Demographic characteristics and particular residence of origin of the examined subjects

Region	East	Northeast	North	Northwest	Southwest	South
n	319	10	85	41	43	42
Age	17.9 ± 0.6	18.3 ± 0.7	17.8 ± 0.7	17.9 ± 0.7	17.9 ± 0.6	17.8 ± 0.5
Gender(F/M)	175/144	6/4	49/36	17/24	22/21	18/24
BMI (kg/m^2^)	20.93 ± 3.1	21.2 ± 3.1	21.2 ± 2.7	20.8 ± 3.4	20.2 ± 3.2	21.9 ± 2.5
Waist (cm)	72.9 ± 8.2	73.3 ± 10.3	73.2 ± 8.4	73.4 ± 8.3	71.0 ± 9.0	75.2 ± 7.2
Province	Zhejiang-16	Liaoning-3	Tianjin-7	Shaanxi-10	Yunnan-14	Hunan-17
	Shanghai-2	Jilin-4	Shandong-14	Shanxi-10	Sichuan-17	Guangxi-16
	Jiangxi-18	Heilongjiang-3	Hubei-10	Qinghai-5	Guizhou-12	Guangdong-9
	Jiangsu-248		Henan-31	Ningxia-1		
	Fujian-13		Hebei-23	Gansu-15		
	Anhui-22

### Sample collection

Examination and sample collection were performed during the first medical screening on admission to the university directly after arrival to HHU using noninvasively collected substrates (urine) in September 2019. Only healthy subjects without chronic diseases were involved in the current investigation to avoid side effects and interactions of diseases on the studied parameters.

### Sample processing

Collection of urine samples (second portion) was performed in the morning using plastic Vacuette® Urine Collection Cups (Greiner Bio-One International AG, Austria).

Evaluation of dimethoate, BPA, and BaP levels in the urine of examinees was performed using liquid chromatography-mass spectrometry (LC-MS). The levels of omethoate and 3-hydroxypyrene, metabolites of dimethoate and BaP and BPA, respectively, were examined. Standard working solutions of omethoate, BPA, and 3-OHBaP (1 μg/mL) were prepared with methanol as the solvent. After continuous dilution 10^4^ times, the standard working solution of 100 pg/mL was obtained. Taking 3-OHBaP as an example, different concentrations of 3-OHBaP standard working solutions were prepared. Fifty microliters of each 3-OHBaP standard working solution was prepared and injected into the system. The collected urine samples (2 mL) were filtered with a 0.22 μM filter membrane, the pH was adjusted to 5.4 by adding acetic acid-sodium acetate buffer (0.5 M), then β-glucuronidase/arylsulfatase (10 μL) and vitamin C (5 mg) were added, and the samples were incubated overnight at room temperature to complete the enzymatic hydrolysis. The samples were extracted after enzymatic hydrolysis by solid-phase extraction with an SPE column (C18 ENVI 0.25 g). The extract was eluted with methanol (2 mL) and dried with nitrogen. Finally, methanol (100 μL) was used to redissolve the analyte to be determined. Fifty microliters of the analyte to be tested was transferred to a liquid chromatography bottle with a microsyringe, which was used specifically for the injection analysis of BaP levels. The detection methods of omethoate and BPA were consistent with those of 3-OHBaP [34].

### Statistical analyses

Statistical treatment of raw data was performed using SPSS 26.0 (IBM Corp., Armonk, NY, USA) software. Geometric mean (GM) values were used as descriptive statistics for pollutant levels. T-tests were used to compare GMs between categories. Multiple regression analysis was performed to specify the association among the pollutant levels, BMI, and region of origin. All models were adjusted for age and sex variability. The results of the tests were considered significant at *P* < 0.05.

## Results

Urine omethoate, BPA, and 3-OHBaP were detected in 100% of the recruited people. The presented results were standardized by volume and creatinine to eliminate the effect of the time of urine collection, urine concentration, and urine flow rate [35].

Hohai University is a multidisciplinary comprehensive university located in Jiangsu Province of East China. The freshmen came from all over the country, including East, North, South, Northeast, Northwest, and Southwest China. We choose East China as the reference category because Hohai University is located in East China, and the environmental exposure levels of subjects may vary considerably according to geography and lifestyle. The obtained data demonstrated that the origin of the students had an important impact on urine chemicals (Table 2). In particular, the volume-based GMs of urine omethoate, BPA, and 3-OHBaP concentrations in students from Southwest China were significantly lower than those in students from East China by 9.49, 10.14, and 8.82%, respectively. Likewise, the standardized GMs of urine omethoate, BPA, and 3-OHBaP concentrations in students from Southwest China were significantly lower than those in students from East China by 10.81, 11.49, and 10.26%, respectively. Data from other regions were more homogenous.

**Table 2:** Weighted geometric means of urinary omethoate, bisphenol A and 3-OHBaP by sex and location among freshmen aged 17–19 years in China

		Total			Males			Females		
Location		Geometric mean	95% confidence interval		Geometric mean	95% confidence interval		Geometric mean	95% confidence interval	
			from	to		from	to		from	to
Urinary omethoate										
Total	μg/L (μg/g)	9.47 (10.80)	9.28 (10.58)	9.67 (11.02)	10.64 (11.11)	10.34 (10.76)	10.95 (11.46)	8.55^‡^(10.53)	8.35 (10.26)	8.74 (10.80)
East China^†^	μg/L (μg/g)	9.38 (10.82)	9.13 (10.52)	9.65 (11.13)	10.84 (11.29)	10.38 (10.78)	11.31 (11.83)	8.49 (10.48)	8.24 (10.12)	8.75 (10.85)
Northeast China	μg/L (μg/g)	9.76 (11.17)	8.00 (9.49)	11.91 (13.15)	11.65 (11.61)	7.50 (7.91)	18.11 (17.03)	8.38 (10.84)	7.32 (8.36)	9.58 (14.04)
North China	μg/L (μg/g)	9.77 (10.98)	9.34 (10.50)	10.22 (11.49)	10.59 (10.79)	9.83 (10.00)	11.40 (11.63)	9.02 (11.16)	8.57 (10.57)	9.50 (11.79)
South China	μg/L (μg/g)	9.96 (11.15)	9.47 (10.67)	10.48 (11.66)	10.48 (11.04)	9.95 (10.48)	11.03 (11.64)	9.14 (11.31)	8.41 (10.43)	9.93 (12.27)
Northwest China	μg/L (μg/g)	9.81 (10.81)	8.92 (9.77)	10.79 (11.96)	10.95 (11.54)	9.71 (10.09)	12.34 (13.21)	8.43 (9.87)	7.44 (8.40)	9.55 (11.59)
Southwest China	μg/L (μg/g)	8.49*(9.65*)	7.82 (8.91)	9.23 (10.45)	9.71 (10.26)	8.45 (8.81)	11.17 (11.95)	7.47 (9.10)	7.01 (8.55)	7.96 (9.67)
Urinary bisphenol A										
Total	μg/L (μg/g)	3.54 (4.04)	3.48 (3.96)	3.61 (4.12)	3.52 (3.68)	3.43 (3.68)	3.62 (3.79)	3.56 (4.39)	3.49 (4.28)	3.65 (4.50)
East China^†^	μg/L (μg/g)	3.55 (4.09)	3.47 (3.98)	3.63 (4.21)	3.56 (3.75)	3.43 (3.59)	3.70 (3.91)	3.54 (4.36)	3.43 (4.22)	3.64 (4.52)
Northeast China	μg/L (μg/g)	3.68 (4.21)	3.17 (3.47)	4.26 (4.11)	3.68 (3.67)	2.66 (2.77)	5.09 (4.86)	3.65 (4.70)	3.01 (3.38)	4.43 (6.53)
North China	μg/L (μg/g)	3.65 (4.11)	3.51 (3.90)	3.80 (4.32)	3.54 (3.57)	3.32 (3.33)	3.78 (3.84)	3.75 (4.64)	3.57 (4.40)	3.95 (4.90)
South China	μg/L (μg/g)	3.65 (4.08)	3.47 (3.87)	3.83 (4.31)	3.50 (3.66)	3.29 (3.44)	3.74 (3.88)	3.86 (4.78)	3.59 (4.45)	4.16 (5.13)
Northwest China	μg/L (μg/g)	3.50 (3.85)	3.23 (3.51)	3.78 (4.23)	3.58 (3.77)	3.21 (3.33)	3.98 (4.26)	3.39 (3.97)	2.99 (3.36)	3.85 (4.69)
Southwest China	μg/L (μg/g)	3.19*(3.62*)	2.98 (3.36)	3.40 (3.89)	3.22 (3.40)	2.85 (2.96)	3.63 (3.90)	3.16 (3.84)	2.94 (3.61)	3.39 (4.08)
Urinary 3-OHBaP										
Total	μg/L (μg/g)	0.34 (0.39)	0.33 (0.38)	0.35 (0.40)	0.38 (0.40)	0.37 (0.39)	0.39 (0.41)	0.31 (0.38)	0.30 (0.37)	0.32 (0.39)
East China^†^	μg/L (μg/g)	0.34 (0.39)	0.33 (0.38)	0.35 (0.40)	0.38 (0.41)	0.37 (0.40)	0.39 (0.42)	0.31 (0.38)	0.30 (0.37)	0.32 (0.39)
Northeast China	μg/L (μg/g)	0.35 (0.40)	0.30 (0.34)	0.41 (0.47)	0.39 (0.39)	0.30 (0.31)	0.52 (0.50)	0.32 (0.41)	0.27 (0.30)	0.39 (0.57)
North China	μg/L (μg/g)	0.35 (0.40)	0.34 (0.38)	0.37 (0.41)	0.38 (0.39)	0.36 (0.36)	0.41 (0.42)	0.33 (0.40)	0.31 (0.38)	0.34 (0.43)
South China	μg/L (μg/g)	0.36 (0.40)	0.34 (0.38)	0.38 (0.42)	0.38 (0.40)	0.35 (0.37)	0.41 (0.42)	0.33 (0.41)	0.31 (0.39)	0.36 (0.44)
Northwest China	μg/L (μg/g)	0.34 (0.38)	0.31 (0.34)	0.38 (0.42)	0.39 (0.41)	0.35 (0.36)	0.43 (0.46)	0.29 (0.34)	0.25 (0.29)	0.33 (0.40)
Southwest China	μg/L (μg/g)	0.31*(0.35*)	0.29 (0.33)	0.33 (0.37)	0.35 (0.37)	0.31 (0.32)	0.39 (0.42)	0.27 (0.33)	0.25 (0.31)	0.29 (0.35)

*Note.* † reference category

* significantly different from the estimate for the reference category (*P* < 0.05)

The numbers in parentheses are creatinine standardized concentrations

The volume-based geometric mean (GM) concentration was 9.47 μg/L (Table 3). The GM of urine omethoate in the female group (8.55 μg/L) was significantly lower than that in the male group (10.64 μg/L). The GM omethoate concentration rose significantly from 9.12 μg/L in individuals with a normal BMI (18.5 ≤ BMI ≤ 23.9) to 14.68 μg/L in individuals with an overweight BMI (BMI > 23.9). However, only a moderate change in GM omethoate concentration was observed between the normal BMI (9.12 μg/L) and the underweight BMI (BMI < 18.5) (8.74 μg/L).

**Table 3:** Weighted geometric means of urinary omethoate, bisphenol A and 3-OHBaP by sex and BMI group among freshmen aged 17–19 years in China

		Total			Males			Females		
BMI group		Geometric mean	95% confidence interval		Geometric mean	95% confidence interval		Geometric mean	95% confidence interval	
			from	to		from	to		from	To
Urinary omethoate										
Total	μg/L (μg/g)	9.47 (10.80)	9.28 (10.58)	9.67 (11.02)	10.64 (11.11)	10.34 (10.76)	10.95 (11.46)	8.55^‡^(10.53^‡^)	8.35 (10.26)	8.74 (10.80)
< 18.5	μg/L (μg/g)	8.74 (10.01)	8.28 (9.48)	9.23 (10.57)	9.66 (10.33)	9.02 (9.66)	10.36 (11.06)	7.88 (9.68)	7.39 (8.86)	8.40 (10.58)
18.5 to 23.9^†^	μg/L (μg/g)	9.12 (10.43)	8.96 (10.23)	9.29 (10.62)	10.19 (10.60)	9.95 (10.32)	10.42 (10.89)	8.30 (10.28)	8.13 (10.01)	8.47 (10.55)
> 23.9	μg/L (μg/g)	14.68*(16.21*)	13.68 (15.13)	15.75 (17.37)	17.49*(18.16*)	16.10 (16.27)	19.00 (20.27)	12.41*(14.54*)	11.68 (13.63)	13.18 (15.51)
Urinary bisphenol A										
Total	μg/L (μg/g)	3.54 (4.04)	3.48 (3.96)	3.61 (4.12)	3.52 (3.68)	3.43 (3.57)	3.62 (3.79)	3.56 (3.79)	3.49 (4.28)	3.65 (4.50)
< 18.5	μg/L (μg/g)	2.93*(3.35)	2.79 (3.15)	3.07 (3.56)	2.83 (3.03)	2.64 (2.83)	3.04 (3.24)	3.03 (3.72)	2.84 (3.41)	3.23 (4.07)
18.5 to 23.9^†^	μg/L (μg/g)	3.50 (4.00)	3.44 (3.91)	3.55 (4.08)	3.47 (3.61)	3.39 (3.51)	3.55 (3.71)	3.52 (4.36)	3.44 (4.24)	3.60 (4.47)
> 23.9	μg/L (μg/g)	4.94*(5.46*)	4.70 (5.14)	5.20 (5.80)	5.13*(5.32*)	4.72 (4.77)	5.57 (5.94)	4.77*(5.59*)	4.49 (5.24)	5.07 (6.00)
Urinary 3-OHBaP										
Total	μg/L (μg/g)	0.34 (0.39)	0.33 (0.38)	0.35 (0.40)	0.38 (0.40)	0.37 (0.39)	0.39 (0.41)	0.31^‡^(0.38^‡^)	0.30 (0.37)	0.32 (0.39)
< 18.5	μg/L (μg/g)	0.27*(0.31)	0.25 (0.29)	0.28 (0.32)	0.29 (0.31)	0.27 (0.29)	0.31 (0.33)	0.25 (0.30)	0.23 (0.28)	0.26 (0.33)
18.5 to 23.9^†^	μg/L (μg/g)	0.34 (0.39)	0.33 (0.38)	0.35 (0.40)	0.38 (0.40)	0.37 (0.39)	0.39 (0.41)	0.31 (0.38)	0.30 (0.37)	0.32 (0.39)
> 23.9	μg/L (μg/g)	0.45*(0.50*)	0.42 (0.46)	0.48 (0.53)	0.52*(0.54*)	0.48 (0.49)	0.57 (0.61)	0.39*(0.45*)	0.37 (0.43)	0.41 (0.48)

*Note.* † reference category

* significantly different from the estimate for the reference category (*P* < 0.05)

‡ significantly different from the estimate for males (*P* < 0.05)

The numbers in parentheses are creatinine standardized concentrations

Males with a BMI greater than 23.9 had a significantly higher GM omethoate concentration (17.49 μg/L) than those with a normal BMI (10.19 μg/L). Likewise, females with a BMI greater than 23.9 had significantly higher GM omethoate concentrations (12.41 μg/L) than those with a normal BMI (8.3 μg/L).

Standardizing omethoate with urinary creatinine concentrations resulted in a GM omethoate concentration of 10.80 μg/g among all recruited people. The standardized GM omethoate concentration in the female group (10.53 μg/g) was significantly lower than that in the male group (11.11 μg/g). The standardized GM omethoate concentration in people with an overweight BMI (16.21 μg/g) was significantly higher than that in people in other BMI groups. The only moderate difference was observed between the normal BMI (10.43 μg/g) and BMI less than 18.5 (10.01 μg/g) groups.

The standardized GM omethoate concentration in overweight males (18.16 μg/g) was significantly higher than that in normal (10.60 μg/g) or underweight males (10.33 μg/g). Likewise, overweight females had significantly higher standardized GM omethoate concentrations (14.54 μg/g) than normal (10.28 μg/g) or underweight females (9.68 μg/g).

The volume-based GM of urine BPA concentration was 3.54 μg/L (Table 3). Interestingly, the GM of urine BPA in the female group (3.56 μg/L) was not significantly different from that in the male group (3.52 μg/L). People with an overweight BMI had a higher GM urine BPA concentration (4.94 μg/L) than those with a normal (3.50 μg/L) or underweight BMI (2.93 μg/L). Notably, a significant change in GM BPA concentration was observed between the normal BMI and the underweight BMI groups.

Males with an overweight BMI had a significantly higher GM BPA concentration (5.13 μg/L) than those with a normal BMI (3.47 μg/L) and an underweight BMI (2.83 μg/L). Likewise, females with an overweight BMI had a significantly higher GM BPA concentration (4.77 μg/L) than those with a normal BMI (3.52 μg/L) and an underweight BMI (3.03 μg/L).

Standardizing BPA according to urinary creatinine concentrations resulted in a GM BPA concentration of 4.04 μg/g for all recruited people. There was no significant difference between the male group (3.68 μg/g) and the female group (4.39 μg/g). The standardized GM BPA concentrations in people with an overweight BMI (5.46 μg/g) were significantly higher than those in individuals in other BMI groups. The only moderate change was observed between the normal BMI (4.00 μg/g) and the underweight BMI (3.35 μg/g) groups.

The standardized GM BPA concentrations in overweight males (5.32 μg/g) were significantly higher than those in normal (3.61 μg/g) or underweight males (3.03 μg/g). Likewise, overweight females had significantly higher standardized GM BPA concentrations (5.59 μg/g) than normal (4.36 μg/g) or underweight females (3.72 μg/g).

The volume-based GM urine BaP concentration was 0.34 ng/L (Table 3). The GM of urine BaP in the female group (0.31 ng/L) was significantly lower than that in the male group (0.38 ng/L). People with overweight BMI had a higher GM urine BaP concentration (0.45 ng/L) than those with a normal (0.34 ng/L) or underweight BMI (0.27 ng/L). Notably, a significant change in GM BaP concentration was also observed between the normal BMI and underweight BMI groups.

Males with an overweight BMI had a significantly higher GM BaP concentration (0.52 ng/L) than those with a normal BMI (0.38 ng/L) and an underweight BMI (0.29 ng/L). Likewise, females with an overweight BMI had a significantly higher GM BaP concentration (0.39 ng/L) than those with a normal BMI (0.31 ng/L) and an underweight BMI (0.25 ng/L).

Standardizing BaP with urinary creatinine concentrations resulted in a GM BaP concentration of 0.39 ng/g for all recruited people. A significant difference was found between the male group (0.40 ng/g) and the female group (0.38 ng/g). The standardized GM BaP concentrations in people with an overweight BMI (0.50 ng/g) were significantly higher than those in individuals in other BMI groups. A significant change was also observed between the normal BMI (0.39 ng/g) and the underweight BMI (0.31 ng/g) groups.

The standardized GM BaP cconcentrations in overweight males (0.54 ng/g) were significantly higher than those in normal (0.40 ng/g) or underweight males (0.31 ng/g). Likewise, overweight females had significantly higher standardized GM oncentrations (0.45 ng/g) than normal (0.38 ng/g) or underweight females (0.30 ng/g).

The association between urinary toxin levels and BMI, as well as the potential confounding effects of age, sex, and waist circumference, was additionally studied in the regression model (Table 4). Particularly, in this regression model, urinary omethoate, BPA, and 3-OHBaP were not associated with age or waist circumference. However, sex and BMI were considered significant predictors of the volume-based concentration of urinary omethoate, BPA, and 3-OHBaP. Notably, male sex was positively associated with the volume-based concentration of urinary omethoate and 3-OHBaP but inversely associated with urinary BPA. However, after standardization according to urinary creatinine, male sex was only inversely associated with urinary BPA. It was also notable that BMI was positively associated with both volume-based and standardized concentrations of urinary omethoate, 3-OHBaP, and BPA.

**Table 4:** Multiple linear regression analysis of the impact of BMI, waist circumference, and sex on urine chemicals in freshmen

Predictor	Unstandardized						Standardized by creatinine					
	omethoate		Bisphenol A		3-OHBaP		omethoate		Bisphenol A		3-OHBaP	
	*β*	*P*	*β*	*P*	*β*	*P*	*β*	*P*	*β*	*P*	*β*	*P*
Sex	1.739	< 0.001*	−0.183	0.001*	0.063	< 0.001*	0.183	0.416	− 0.880	< 0.001*	0.007	0.394
Age	0.011	0.923	−0.013	0.734	−0.001	0.797	−0.096	0.535	−0.060	0.278	−0.005	0.317
BMI	0.572	< 0.001*	0.170	< 0.001*	0.016	< 0.001*	0.588	< 0.001*	0.172	< 0.001*	0.016	< 0.001*
Waist	0.007	0.648	0.004	0.476	7.743E-5	0.877	0.010	0.647	0.005	0.477	0.000224	0.756

*Note.* * significantly different from estimate for the reference category

## Discussion

In this study, we chose 3 kinds of very common pollutants in the Chinese environment to represent the exposure level of pollutants in young Chinese people aged 17 ~ 19 years. In addition, the detection did not rely on a blood sample but rather urinary samples obtained from noninvasive sources, which are easily obtained and inexpensive. Urine could also better reflect the changes in human metabolism because the metabolite concentration is higher in urine than in human plasma or serum [36]. In order to reflect recent exposure levels and avoid changes in concentration caused by chemical metabolism, we collected samples when the students entered school for the first time.

The obtained data demonstrate that the freshmen of Hohai University originating from distinct geographic regions of China are characterized by high pollutant exposure levels. Bushnik, T. reported that the urinary level of BPA in Canada (1.16 μg/L) was almost 1/3 of that in our sample (3.54 μg/L) [37]. This difference may reflect the differences in the situation of BPA pollution between the Chinese and other countries’ environments. Yu et al. reported that urinary 1-OHP concentrations increased with increasing air concentrations of BaP in an industrial area in Lanzhou City [38]. Notably, there are no data reports on the urinary level of dimethoate in healthy adults in other countries. Therefore, it is meaningful to detect the baseline level of these three pollutants to reflect the effects of the local environment on the human body. Furthermore, males showed a significantly higher urinary level of BPA than females in Canada, while no significant sex difference in BPA levels was observed in our data. This difference may reflect the differences in pharmacokinetic factors between sexes and races, the relevance of which is not currently known [39].

Despite no differences in urinary BPA between sexes in our data, males exhibited a significantly higher level of urinary omethoate and 3-OHBaP than females (Table 3). Moreover, sex was considered a significant predictor of the urinary level of omethoate and 3-OHBaP in the regression model. This finding may reflect the differences in lifestyle between males and females.

The association of BMI and the urinary levels of omethoate, 3-OHBaP, and BPA was additionally studied in regression models. In particular, BMI could be considered a significant predictor of the urinary level of these three pollutants regardless of whether the values are standardized by urinary creatinine (Table 4). Since most persistent organic pollutants (POPs) are lipophilic, it has been widely shown that POPs can be stored in adipose tissue [40–42]. Moreover, the accumulated POPs could increase the risks of obesity and diabetes by inducing adipogenesis [43, 44] and inhibiting glucose uptake [45]. This indicates that adipose tissue can act as a storage for most pollutants in our bodies. The stored pollutants in adipose tissue can further enhance adipogenesis and insulin resistance. Our data and previous findings indicate that increasing BMI and obesity can be risk factors for greater accumulation of pollutants in the human body, which could further act as obesogens.

In addition to differences according to BMI, we found that the students from Southwest China had a significantly lower level of all three pollutants than those from East China, which was considered the control group (Table 2). Interestingly, there was no significant difference in BMI between students from Southwest China and those from East China. This finding reflects that the living environment and lifestyle may determine this difference [46]. Although there are very few reports on the differences among various provinces in China, it has been reported that air pollution in North China is much worse than that in South China [47]. This may partially explain our finding and indicates that the living environment can determine the level of accumulated pollutants in our bodies. To investigate the relationship between the accumulated pollutants in the human body and health, further research is needed to understand the detailed differences in the living environment and lifestyle between people from Southwest China and other geographic regions in China.

Some studies have reported that urinary levels of these chemicals may have strong correlations with some adverse health effects as well as the studies reported in CDC. Wang et al. [48] reported that preconception concentrations of BPA in female urine were associated with decreased fecundability, particularly among older women. In addition, Niu et al. [49] reported that occupational BaP exposure may reduce coke oven workers’ neurobehavioral function and monoamine, amino acid and choline neurotransmitter levels. However, few studies have assessed the associations between urinary dimethoate levels and adverse health effects. This study contributes to our understanding of the baseline dimethoate, BaP, and BPA levels in healthy adults and will help to improve public health awareness and have important implications for health policy formulation.

## Data Availability

All data generated or analyzed during this study are included in this published article.

## Abbreviations

BaP: Benzo(a)pyrene
BPA: Bisphenol A
GMs: Geometric means
BMI: Body mass index
PAHs: Polycyclic aromatic hydrocarbons
IARC: International Agency for Research on Cancer
HHU: Hohai University
LC-MS: Liquid chromatography-mass spectrometry
POPs: persistent organic pollutants
